# Comprehensive Genomic Characterization of Marine Bacteria *Thalassospira* spp. Provides Insights into Their Ecological Roles in Aromatic Hydrocarbon-Exposed Environments

**DOI:** 10.1128/spectrum.03149-22

**Published:** 2022-10-03

**Authors:** Go Kayama, Robert A. Kanaly, Jiro F. Mori

**Affiliations:** a Graduate School of Nanobiosciences, Yokohama City University, Yokohama, Japan; University of Minnesota

**Keywords:** aromatic hydrocarbons, biodegradation, genomics, marine bacteria

## Abstract

The marine bacterial genus *Thalassospira* has often been identified as an abundant member of polycyclic aromatic hydrocarbon (PAH)-exposed microbial communities. However, despite their potential usability for biotechnological applications, functional genes that are conserved in their genomes have barely been investigated. Thus, the goal of this study was to comprehensively examine the functional genes that were potentially responsible for aromatic hydrocarbon biodegradation in the *Thalassospira* genomes available from databases, including a new isolate of *Thalassospira*, strain GO-4, isolated from a phenanthrene-enriched marine bacterial consortium. Strain GO-4 was used in this study as a model organism to link the genomic data and their metabolic functions. Strain GO-4 growth assays confirmed that it utilized a common phenanthrene biodegradation intermediate 2-carboxybenzaldehyde (CBA) as the sole source of carbon and energy, but did not utilize phenanthrene. Consistently, strain GO-4 was found to possess homologous genes of *phdK*, *pht*, and *pca* that encode enzymes for biodegradation of CBA, phthalic acid, and protocatechuic acid, respectively. Further comprehensive genomic analyses for 33 *Thalassospira* genomes from databases showed that a gene cluster that consisted of *phdK* and *pht* homologs was conserved in 13 of the 33 strains. *pca* gene homologs were found in all examined genomes; however, homologs of the known PAH-degrading genes, such as the *pah*, *phn*, or *nah* genes, were not found. Possibly *Thalassospira* spp. co-occupy niches with other PAH-degrading bacteria that provide them with PAH degradation intermediates and facilitated their inhabitation in PAH-exposed microbial ecosystems.

**IMPORTANCE** Comprehensive investigation of multiple genomic data sets from targeted microbial taxa deposited in databases may provide substantial information to predict metabolic capabilities and ecological roles in different environments. This study is the first report that details the functional profiling of *Thalassospira* spp. that have repeatedly been found in polycyclic aromatic hydrocarbon (PAH)-exposed marine bacterial communities by using genomic data from a new isolate, *Thalassospira* strain GO-4, and other strains in databases. Through screening of functional genes potentially involved in aromatic hydrocarbon biodegradation across 33 *Thalassospira* genomes and growth assays for strain GO-4, it was suggested that *Thalassospira* spp. unexceptionally conserved the ability to metabolize single-ring, PAH biodegradation intermediates, while being incapable of utilizing PAHs. This expanded our understanding of this potentially important contributing member to PAH-degrading microbial ecosystems; such species are considered to be specialized in driving downstream reactions of PAH biodegradation.

## INTRODUCTION

The environmental fate of toxic and persistent organic pollutants that are released from anthropogenic activities has been of great concern. In marine environments, indigenous microorganisms play central roles in global carbon and nutrient cycling and thus are expected to be main drivers of restoration of natural environments when they are exposed to an accidental release of organic pollutants—such as in ocean oil spill disasters (1). Microbial function genes that were characterized as responsible for key steps in the biotransformation of specific hydrocarbons have been employed as functional biomarkers to evaluate the ability of microorganisms for removal of organic pollutants (2–4). At the same time, microbial (meta)genomic research has contributed to understanding complex microbial ecosystems that develop in polluted environments through identification of microbial taxa that play central roles in pollutant biodegradation and that may be applied to bioremediation (5–7).

The alphaproteobacterial genus *Thalassospira* (*Rhodospirillales*, *Thalassospiraceae*) was first characterized in 2002 (8), has been discovered globally in marine environments, and has repeatedly been identified as an abundant member of polycyclic aromatic hydrocarbon (PAH)-exposed marine microbial communities (9–12). Additionally, there are a few examples of isolates that were claimed to be capable of degrading PAHs and potentially growing on them as the carbon source (13–15). However, despite such unique metabolic capabilities and potential high usability for biotechnological applications, genomic information of *Thalassospira* strains has not been adequately discussed and characterized. Other well-studied bacterial groups that are capable of biodegrading and growing on aromatic hydrocarbons, such as Mycobacterium, Pseudomonas, or sphingomonad strains, are known to have acquired and conserved the specialized functional genes in their genomes that are responsible for their metabolisms: e.g., aromatic ring-hydroxylating dioxygenases (EC 1.14.12.x) and aromatic ring cleavage dioxygenases (EC 1.13.11.x) (16–20). In contrast, in *Thalassospira*, it remains largely unknown how these functional genes have been conserved evolutionally in different strains and how distinct they are from those in the known aromatic hydrocarbon-degrading bacterial groups.

In this study, comprehensive investigation of the genomes from *Thalassospira* spp. that were available in public databases was performed for the first time, in which the functional genes that were potentially involved in aromatic hydrocarbon biodegradation were screened across 33 *Thalassospira* genomes. Additionally, the complete genome of a new *Thalassospira* isolate, strain GO-4, which was obtained from a phenanthrene-enriched marine bacterial consortium (21), was sequenced and this organism was used as a model to link genomic data and metabolic functions. Through these investigations, this study aimed to predict the metabolic capability conserved in *Thalassospira* spp. and to understand their ecological roles in microbial ecosystems that drive aromatic hydrocarbon biodegradation.

## RESULTS

### Isolation of strain GO-4, which grows on CBA as the sole carbon source.

**FIG 1:**
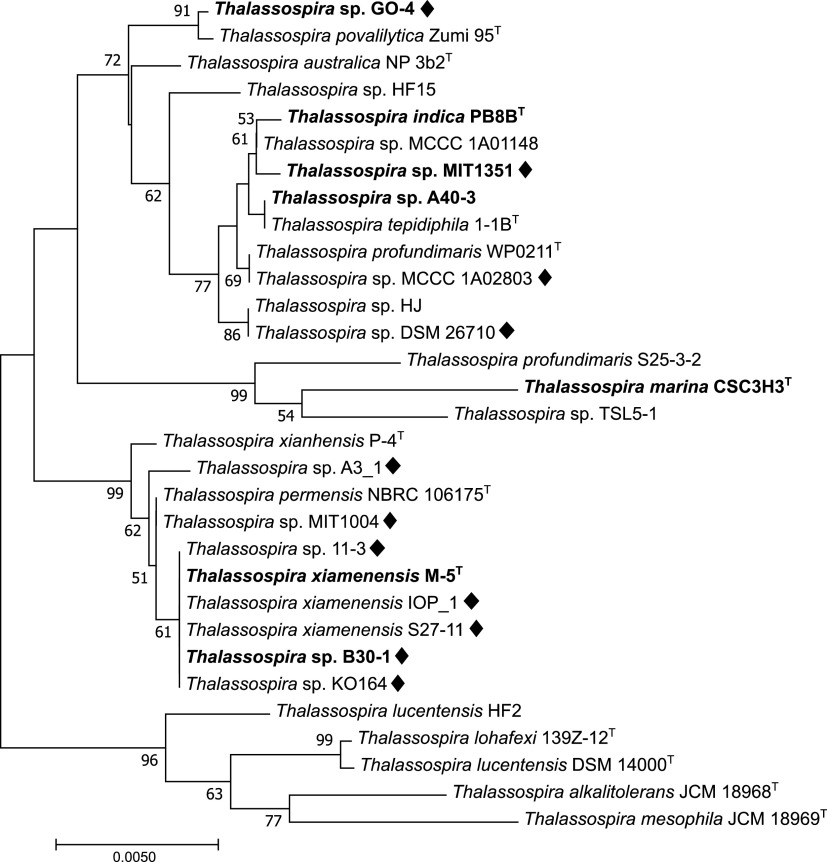
Neighbor-joining phylogenetic tree of 16S rRNA genes of strain GO-4 and reference *Thalassospira* strains. Strains with complete genomes available in databases are shown in boldface. Black diamonds indicate strains that possess the *phdK*-*pht* gene cluster in their genomes. The tree was created with 1,000 bootstrap iterations, and values below 50% are not reported.

**FIG 2:**
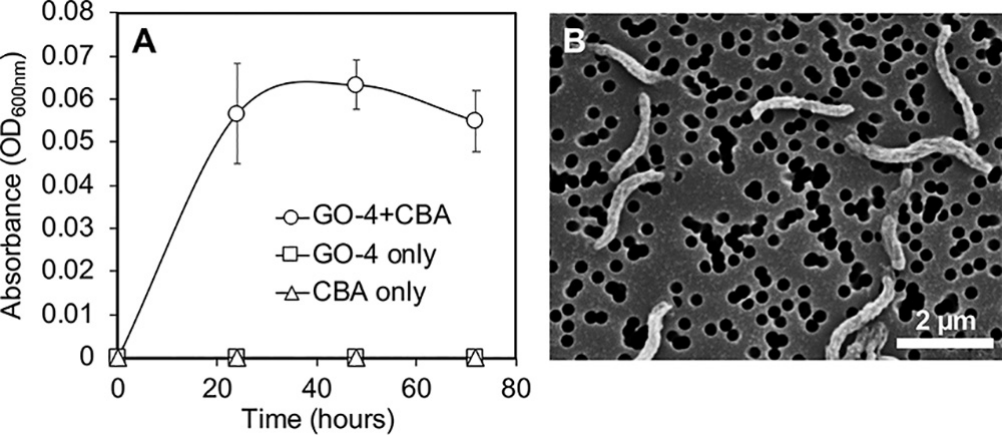
(A) Growth monitoring of strain GO-4 that grew on 100 mg L^−1^ 2-carboxybenzaldehyde (CBA) as the sole carbon source. OD_600_ values of the cultures that contained bacterial cells with CBA (○; *n* = 3), bacterial cells only (□; *n* = 1), or CBA only (△; *n* = 1) are shown. (B) Scanning electron microscopic image of strain GO-4 cells that grew on CBA for 5 days.

*Thalassospira* sp. strain GO-4 was isolated from a phenanthrene-enriched coastal marine bacterial consortium that has been maintained in the laboratory (see Materials and Methods and reference 21). Taxonomic analysis based on 16S rRNA gene sequencing indicated that strain GO-4 was most closely related to Thalassospira povalilytica strain Zumi 95^T^ (99.93% identity) (Fig. 1), a polyvinyl alcohol-degrading organism that was isolated from Tokyo Bay (22). Although strain GO-4 was isolated from a phenanthrene-grown consortium, the isolate did not exhibit clear growth on phenanthrene or 1-hydroxy-2-naphthoic acid, a typically biodegraded downstream product of phenanthrene biodegradation (23), as a sole carbon source. When strain GO-4 cells were incubated with the putative further downstream compound of phenanthrene biodegradation, 2-carboxybenzaldehyde (CBA), cells showed an apparent increase in cell density, reaching stationary phase after around 24 h (Fig. 2A), and the spiral-rod-shaped cells multiplied in the culture (Fig. 2B).

### The complete genome of strain GO-4 and taxonomic characterization.

**FIG 3:**
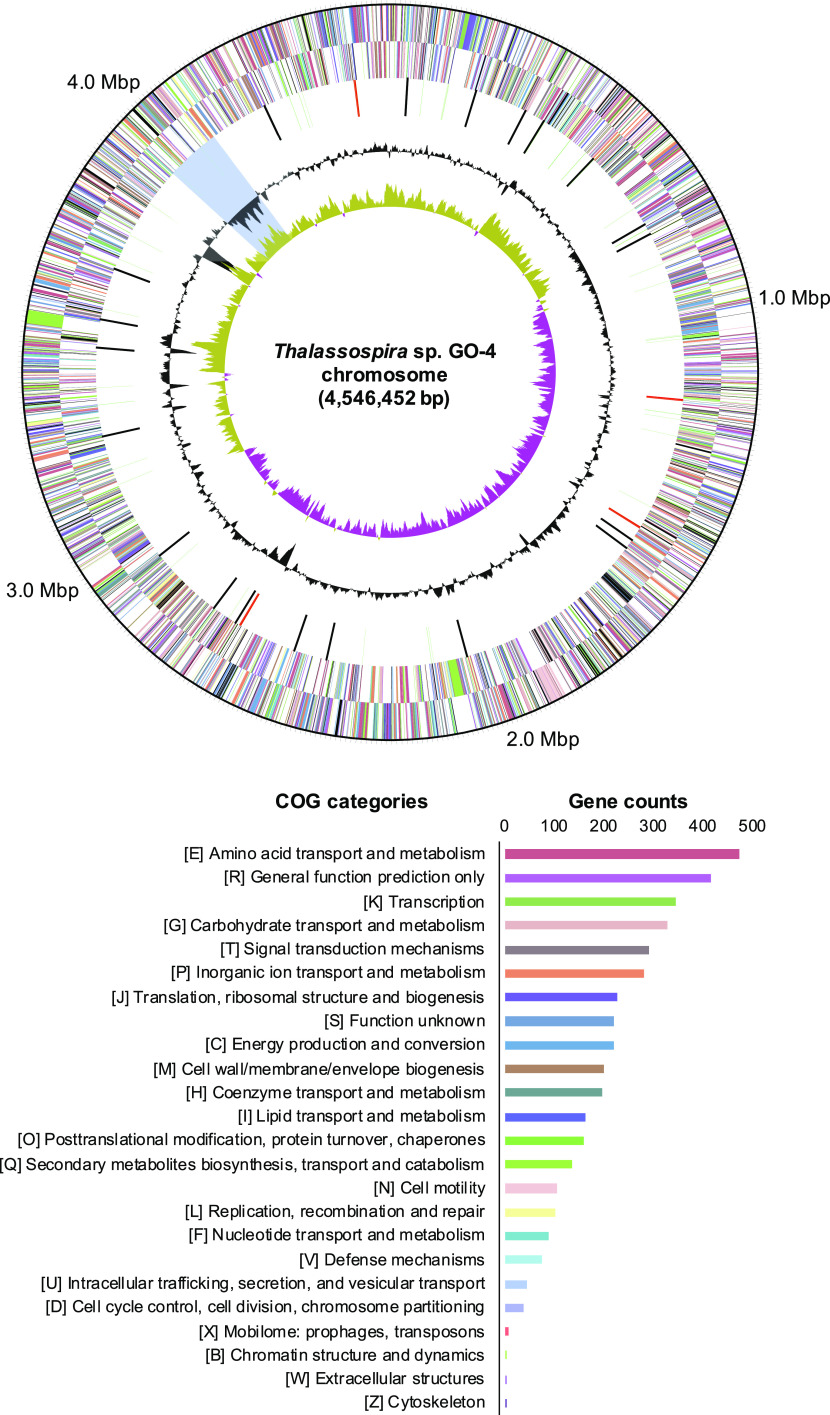
Circular map of the strain GO-4 chromosome with functional annotation. Rings from outside to the center represent genes on the forward strand and reverse strand (colored by COG annotation categories, as listed in the bar graph with gene counts), RNA genes (tRNAs, green; rRNAs, red; other RNAs, black), GC content (gray), and GC skew (yellow and purple). The putative foreign gene-rich region with lower GC contents is shown with a blue background.

The complete genome sequence of strain GO-4 was successfully obtained through the hybrid assembly technique using the short-read (DNBSEQ) and long-read (GridION) sequencing data (see Table S1 in the supplemental material) (21). The genome of strain GO-4 consisted of a single circular chromosome with a size of 4,546,452 bp without plasmids (Fig. 3), and 4,046 coding genes (CDSs) were detected on the chromosome (according to the Prokaryotic Genome Annotation Pipeline [PGAP]). The COG categorization of these coding genes and their location on the chromosome are summarized in Fig. 3. The genome of strain GO-4 showed 97.7% average nucleic acid identity (ANI) to *T. povalilytica* Zumi 95^T^ (draft genome; 21 contigs), 84.0% ANI to Thalassospira indica PB8B^T^ (complete genome; 1 chromosome), 83.9% ANI to Thalassospira profundimaris WP0211^T^ (draft genome; 28 contigs), and 83.0% ANI to Thalassospira australica NP 3b2^T^ (draft genome; 32 contigs).

### Functional genes of aromatic hydrocarbon degradation conserved in *Thalassospira* genomes.

As the potential marker genes responsible for aromatic hydrocarbon biodegradation, genes that were annotated as homologous genes of the known aromatic ring-hydroxylating dioxygenase genes or aromatic ring cleavage dioxygenase genes were screened in the genomes of strain GO-4 and the selected 32 *Thalassospira* strains in databases. Strain GO-4 and other 12 strains possessed homologous genes that encode the subunits of phthalate 4,5-dioxygenase Pht2/Pht3 (EC 1.14.12.7) in their genomes. However, genes that were annotated as other known aromatic ring-hydroxylating dioxygenase genes (EC 1.14.12.x) were not found in all genomes (Table 1). Regarding aromatic ring cleavage dioxygenase genes, all the selected 33 genomes possessed homologous genes for alpha/beta subunits of protocatechuate 3,4-dioxygenase PcaGH (EC 1.13.11.3), while genes for other known aromatic ring cleavage dioxygenases (EC 1.13.11.x) were not found except for catechol 2,3-dioxygenase XylE (EC 1.13.11.2) (Table 1); *xylE* was found in four reference genomes, although other *xyl* genes responsible for the downstream reactions of catechol degradation were not.

**TABLE 1:** List of the representative known functional gene products for aromatic hydrocarbon degradation and number of the selected 33 *Thalassospira* genomes that were found to possess each gene

Gene product	No. of *Thalassospira* genomes that have gene encoding the product shown
Aromatic ring-hydroxylating dioxygenase	
Benzene 1,2-dioxygenase (EC 1.14.12.3)	0/33
Phthalate 4,5-dioxygenase (EC 1.14.12.7)	13/33
Benzoate 1,2-dioxygenase (EC 1.14.12.10)	0/33
Toluene 2,3-dioxygenase (EC 1.14.12.11)	0/33
Naphthalene 1,2-dioxygenase (EC 1.14.12.12)	0/33
Biphenyl 2,3-dioxygenase (EC 1.14.12.18)	0/33
Aromatic ring cleavage dioxygenase	
Catechol 1,2-dioxygenase (EC 1.13.11.1)	0/33
Catechol 2,3-dioxygenase (EC 1.13.11.2)	4/33
Protocatechuate 3,4-dioxygenase (EC 1.13.11.3)	33/33
Protocatechuate 4,5-dioxygenase (EC 1.13.11.8)	0/33

**FIG 4:**
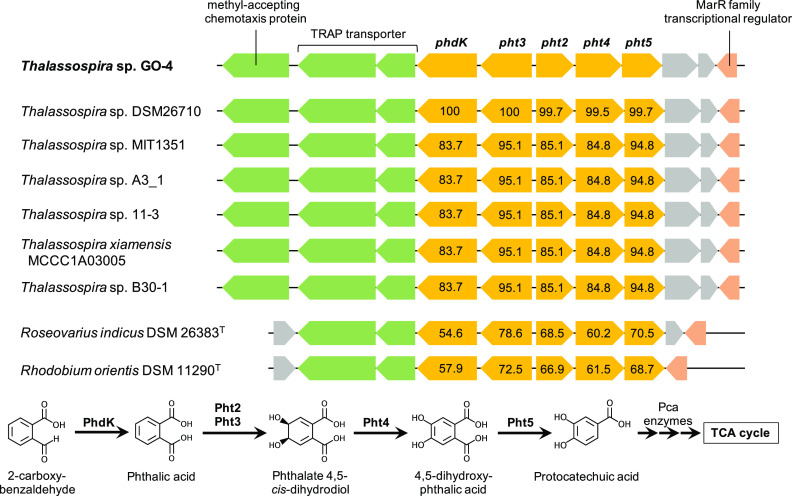
*phdK-pht* gene cluster that is potentially responsible for 2-carboxybenzaldehyde biotransformation and is conserved in the genomes of *Thalassospira* strains, Roseovarius indicus DSM 26383^T^, and Rhodobium orientis DSM 11290^T^. Amino acid sequence similarities (percentages) of PhdK and Pht enzymes to those of strain GO-4 are presented.

The *pht2/pht3* gene homologs in the strain GO-4 genome were present as a putative *pht* gene cluster (Fig. 4), that shall be responsible for biotransformation of phthalic acid to protocatechuic acid (24, 25), and the *pcaGH* homologs were also found to be clustered with other *pca* genes that shall catalyze the ring cleavage of protocatechuic acid and downstream degradation to the tricarboxylic acid (TCA) cycle (26, 27) (Table 2). Degradation of phthalic acid shall be initiated from the addition of two oxygen atoms to the benzene ring by phthalate 4,5-dioxygenase Pht2/Pht3 and phthalate 4,5-*cis*-dihydrodiol dehydrogenase Pht4 (EC 1.3.1.64), and then subsequently decarboxylated by 4,5-dihydroxyphthalate decarboxylase Pht5 (EC 4.1.1.55) resulting in the generation of protocatechuic acid (Fig. 4). Furthermore, a gene that was located next to *pht* genes in the strain GO-4 genome was annotated as the *phdK* gene, which encodes 2-carboxybenzaldehyde dehydrogenase (EC 1.2.1.78) (28). This putative *phdK-pht* gene cluster, including the neighboring genes that potentially encode a methyl-accepting chemotaxis protein, tripartite ATP-independent periplasmic (TRAP) transporter—known to typically be involved in phthalate uptake (29)—and a MarR family transcriptional regulator, were found to be conserved in genomes of other *Thalassospira* strains available in databases (Fig. 4 and Table 3), throughout different phylogenetic branches (Fig. 1). The amino acid sequences of these putative PhdK and Pht enzymes showed 83 to 100% identities among *Thalassospira* strains, and the enzymes in *Thalassospira* sp. strain DSM26710 were most closely related to those in strain GO-4. This gene set was also found in the genomes of Roseovarius indicus DSM 26383^T^ (IMG genome ID 2619618997; 54 to 79% amino acid identities), a *Rhodobacteraceae* bacterium that was isolated from a PAH-degrading marine bacterial consortium (30), and Rhodobium orientis DSM 11290^T^ (IMG genome ID 2887621230; 57 to 73% amino acid identities), a phototrophic bacterium that was isolated from coastal seawater (31) (Fig. 4). Considering downstream metabolic processes, an aromatic ring cleavage dioxygenase, protocatechuate 3,4-dioxygenase PcaGH, and other Pca enzymes that are putatively encoded by the clustered *pca* gene homologs in the strain GO-4 genome should be responsible for biodegradation of protocatechuic acid into the TCA cycle. The *pca* gene cluster was found in all 33 *Thalassospira* genomes in the databases (Table 3).

**TABLE 2:** List of functional genes that were found as the two gene clusters in the strain GO-4 genome and are responsible for biodegradation of 2-carboxybenzaldehyde via phthalic acid and protocatechuic acid

Gene, product	EC no.	COG category	aa length	Locus	IMG gene ID
*phdK*, 2-carboxybenzaldehyde dehydrogenase	1.2.1.78	C	492	4025393–4026871	2963527656
*pht3*, phthalate 4,5-dioxygenase oxygenase subunit	1.14.12.7	P	425	4023933–4025210	2963527655
*pht2*, phthalate 4,5-dioxygenase reductase subunit	1.14.12.7	C	316	4022846–4023796	2963527654
*pht4*, phthalate 4,5-*cis*-dihydrodiol dehydrogenase	1.3.1.64	R	382	4021639–4022862	2963527653
*pht5*, 4,5-dihydroxyphthalate decarboxylase	4.1.1.55		330	4020650–4021642	2963527652
*pcaB*, 3-carboxy-*cis*,*cis*-muconate cycloisomerase	5.5.1.2	F	445	1138719–1140056	2963525014
*pcaF*, acetyl-CoA C-acyltransferase	2.3.1.16	I	401	1140209–1141414	2963525015
*pcaJ*, 3-oxoadipate CoA-transferase beta subunit	2.8.3.6	I	257	1141438–1142211	2963525016
*pcaI*, 3-oxoadipate CoA-transferase alpha subunit	2.8.3.6	I	284	1142226–1143080	2963525017
*pcaG*, protocatechuate 3,4-dioxygenase alpha subunit	1.13.11.3	Q	195	1143223–1143810	2963525018
*pcaH*, protocatechuate 3,4-dioxygenase beta subunit	1.13.11.3	Q	233	1143814–1144515	2963525019
*pcaC*, 4-carboxymuconolactone decarboxylase	4.1.1.44	R	130	1144521–1144913	2963525020
*pcaD*, 3-oxoadipate enol-lactonase	3.1.1.24	H	266	1144906–1144706	2963525021

**TABLE 3:** Information of selected *Thalassospira* genomes in the databases

Organism	IMG genome ID	NCBI accession no.	Completeness (no. of contigs)	Presence of:	
				*phdK-pht* cluster	*pca* cluster
*Thalassospira* sp. strain GO-4	2963523905	ASM2358896	Complete (1)	+	+
Thalassospira povalilytica Zumi 95^T^		ASM284423	Draft (21)		+
Thalassospira australica NP3b2^T^	2617271273	ASM76329	Draft (32)		+
*Thalassospira* sp. strain HF15	2895203324	ASM1180652	Draft (25)		+
Thalassospira indica PB8B^T^	2840662270	ASM340309	Complete (1)	−	+
*Thalassospira* sp. strain MCCC 1A01148	2724679112	ASM161379	Draft (24)		+
*Thalassospira* sp. strain MIT1351	2681813577		Complete (1)	+	+
*Thalassospira* sp. strain A40-3		ASM1587148	Complete (1)	−	+
Thalassospira tepidiphila 1-1B^T^	2829832560	ASM1192768	Draft (5)		+
Thalassospira profundimaris WP0211^T^	2529292925	ASM30027	Draft (28)		+
*Thalassospira* sp. strain MCCC 1A02491	2724679110	ASM161378	Draft (33)	+	+
*Thalassospira* sp. strain MCCC 1A02803		ASM198342	Draft (22)	+	+
*Thalassospira* sp. strain HJ	2648501770	ASM94841	Draft (22)		+
*Thalassospira* sp. strain DSM 26710	2728369743	ASM361021	Draft (12)	+	+
Thalassospira profundimaris S25-3-2	2808606712	ASM332675	Draft (85)		+
Thalassospira marina CSC3H3^T^	2775506933	ASM284437	Complete (2)	−	+
*Thalassospira* sp. strain TSL5-1		ASM190769	Draft (27)		+
Thalassospira xianhensis P-4^T^		ASM332647	Draft (100)		+
*Thalassospira* sp. strain A3_1	2893749381	ASM1774421	Draft (11)	+	+
Thalassospira permensis NBRC 106175^T^	2585427703		Draft (72)		+
*Thalassospira* sp. strain MIT1004	2839174240	ASM185854	Draft (47)	+	+
*Thalassospira* sp. strain 11-3	2744054425	ASM320199	Draft (28)	+	+
Thalassospira xiamenensis M-5^T^	2681813519	ASM30023	Complete (2)	−	+
Thalassospira xiamenensis IOP_1		ASM2102127	Draft (65)	+	+
Thalassospira xiamenensis S27-11		ASM332671	Draft (56)	+	+
Thalassospira xiamenensis MCCC 1A03005	2744054535	ASM161816	Draft (62)	+	+
*Thalassospira* sp. strain B30-1		ASM1576757	Complete (1)	+	+
*Thalassospira* sp. strain KO164	2751185779		Draft (2)	+	+
Thalassospira lucentensis HF2	2915113161	ASM1180642	Draft (13)		+
Thalassospira lohafexi 139Z-12^T^		ASM284427	Draft (23)		+
Thalassospira lucentensis DSM 14000^T^	2526164517	ASM42126	Draft (22)		+
Thalassospira alkalitolerans JCM 18968^T^	2791355065	ASM211574	Draft (72)		+
Thalassospira mesophila JCM 18969^T^	2791355063	ASM211575	Draft (94)		+

### The *phdK-pht* gene cluster was located on a foreign gene-rich region of the strain GO-4 genome.

**FIG 5:**
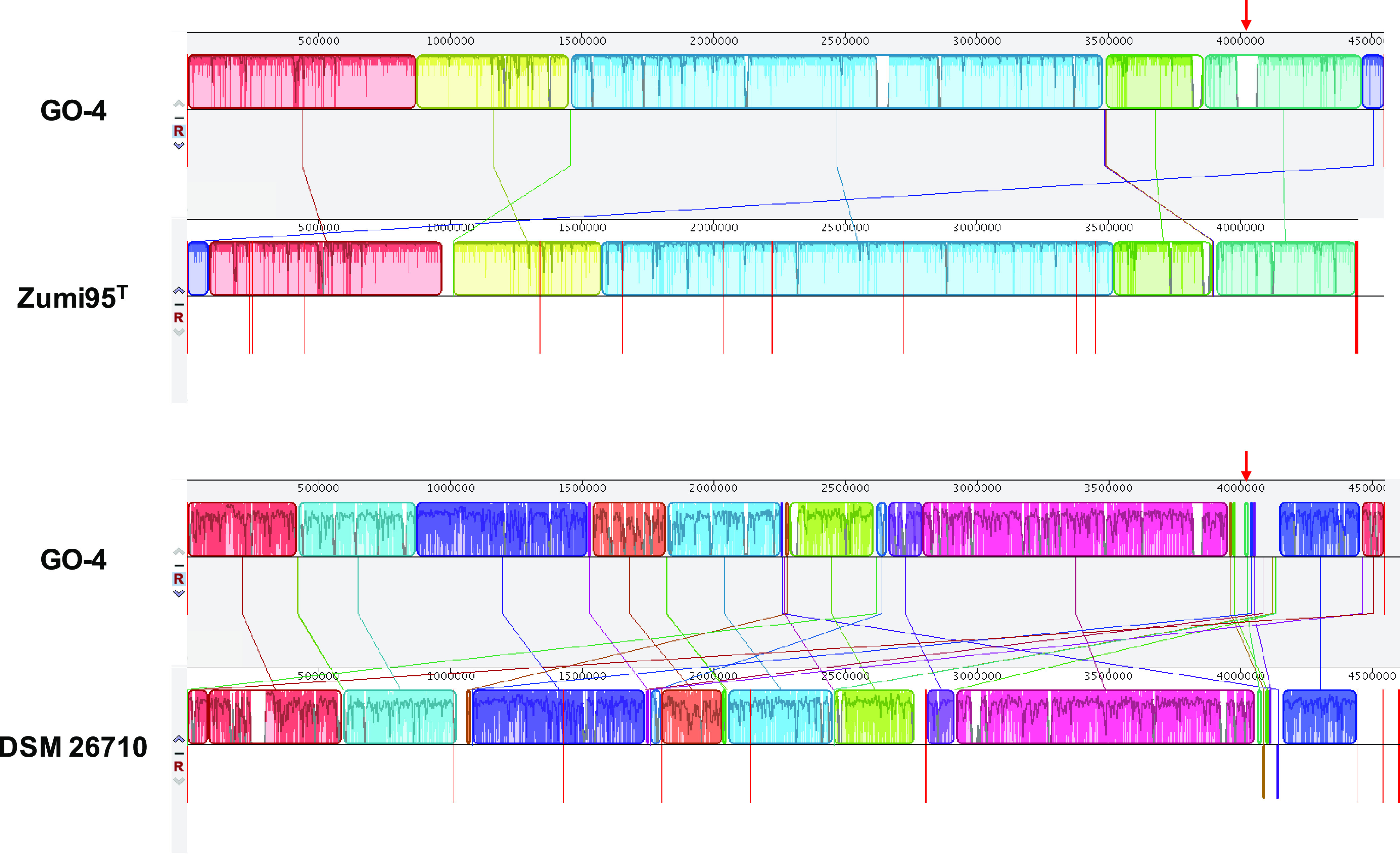
Sequence alignment comparisons of the strain GO-4 genome against the genomes of *T. povalilytica* Zumi 95^T^ (above) and *Thalassospira* sp. strain DSM 26710 (below). Red arrows indicate the location of *phdK-pht* gene cluster that was located on the putative foreign gene-rich genomic region.

Sequence alignment comparison between genomes of strain GO-4 and the closest strain, *T. povalilytica* Zumi 95^T^, indicated that the lack of the *phdK-pht* gene cluster in the strain Zumi95^T^ draft genome was not due to sequencing gaps between different contigs (Fig. 5). In fact, the *phdK-pht* gene cluster of strain GO-4 was located on the less-conserved genomic region (approximately genome positions 3.98 to 4.06 Mb) (Fig. 5), which showed lower GC content than other regions (Fig. 3), suggesting that this region was inserted and enriched with horizontally acquired genes (32, 33). This region also included functional gene operons for nitrate reductase, nitric oxide reductase, isoquinoline oxidoreductase, and ribose transport systems (Table S2). Through further alignment comparison against the genome of strain DSM 26710, which possessed the *phdK-pht* gene cluster most closely related to that of strain GO-4 (Fig. 4), the *phdK-pht* gene cluster and surrounding operons were found to be distributed at different locations and in different orientations in the strain DSM 26710 genome, which may indicate that these operons were individually mobilized and transposed (Fig. 5).

### Identification of biotransformation products of CBA by liquid chromatography-electrospray ionization tandem mass spectrometry.

**FIG 6:**
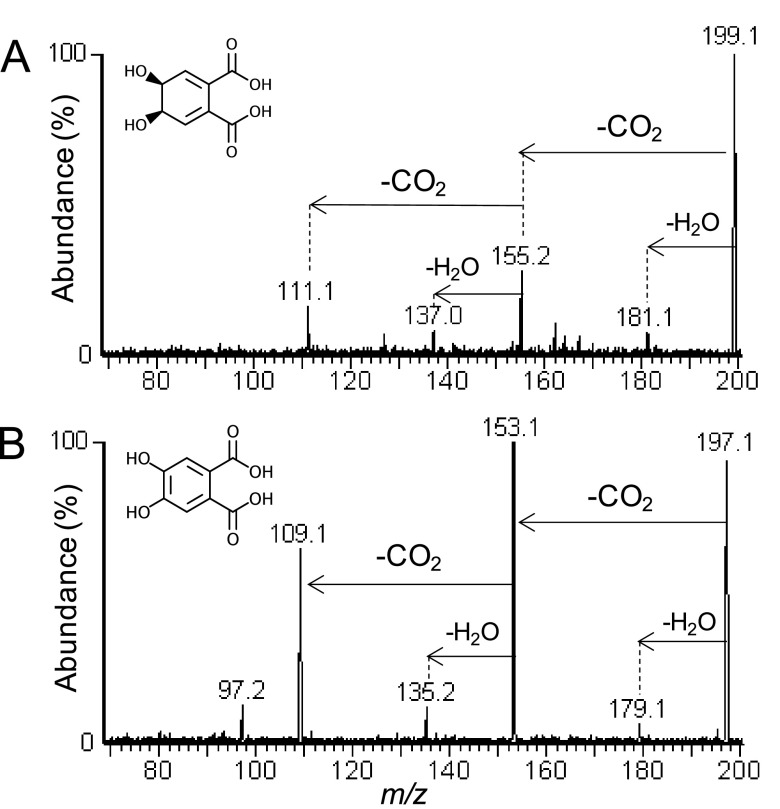
LC-ESI(−)-MS/MS product ion scan mass spectra of the biotransformation products that corresponded to (A) [M – H]^−^ = 199, which was assigned as phthalate 4,5-*cis*-dihydrodiol, and (B) [M – H]^−^ = 197, which was assigned as 4,5-dihydroxyphthalic acid.

When strain GO-4 was grown on CBA as the sole carbon source, two biodegradation products were detected by liquid chromatography-electrospray ionization tandem mass spectrometry [LC-ESI(−)-MS/MS] analyses that corresponded to the deprotonated molecules [M – H]^−^ = 199 and [M – H]^−^ = 197. As shown in Fig. 6A and B, results of product ion scan analyses of these compounds revealed similar mass spectra in that two consecutive losses of 44 Da each as CO_2_ were detected as the most abundant fragments during collision-induced dissociation (CID) of both compounds. In the case of the compound that corresponded to [M – H]^−^ = 199, *m/z* 155 and *m/z* 111 were observed (Fig. 6A). The fragment *m/z* 181 represented a loss of water as 18 Da from the parent deprotonated molecule and the fragment *m/z* 137 represented losses of CO_2_ and water (Fig. 6A). When these results were considered in combination with a molar mass of 200 Da and a molecular formula of C_8_H_8_O_6_, they provided evidence that this compound was a phthalate-dihydrodiol. As shown in Fig. 6B, results of CID of the compound that corresponded to [M – H]^−^ = 197 revealed losses of 44 Da (CO_2_) and 88 Da (2CO_2_) from the parent deprotonated molecule as *m/z* 153 and *m/z* 109, respectively; losses of water occurred identically to those described above. Taken together with a molar mass of 198 and a molecular formula of C_8_H_6_O_6_, these results provided evidence that this compound was a dehydroxylated phthalic acid. Shown in Fig. 6 are the compounds phthalate 4,5-*cis*-dihydrodiol (Fig. 6A) and 4,5-dihydroxyphthalic acid (Fig. 6B), which were reported as the biotransformation products of phthalic acid by the enzymes Pht2/Pht3 and Pht4 (Fig. 4) (25). When strain GO-4 was incubated with a mixture of 100 mg L^−1^ each of CBA and phenanthrene, phenanthrene loss was not detected after 8 days of incubation, which confirmed that strain GO-4 was unable to biodegrade this compound, even though cells grew by consuming CBA (Fig. S1).

## DISCUSSION

### The putative CBA-degrading gene cluster that was conserved in *Thalassospira*.

2-Carboxybenzaldehyde (CBA), phthalic acid, and protocatechuic acid are known as the intermediate products in one of the typical phenanthrene biodegradation pathways, the so-called “phthalate pathway,” which was previously reported in both Gram-negative (Pseudomonas spp.) and Gram-positive (Mycobacterium and *Nocardioides* spp.) bacteria (23, 34, 35). The key reactions for the phthalate pathway shall be the transformation of 1-hydroxy-2-naphthoic acid to phthalic acid via CBA, instead of generating 1,2-dihydroxynaphthalene that is further degraded via salicylic acid (the so-called “salicylate pathway”). Functional genes that are responsible for those key reactions were previously characterized in Mycobacterium and *Nocardioides* spp. as the *phd* gene cluster: 1-hydroxy-2-naphthoate dioxygenase (PhdI; EC 1.13.11.38) and *cis*-2′-carboxybenzalpyruvate aldolase (PhdJ; EC 4.1.2.34) convert 1-hydroxy-2-naphthoic acid to CBA, which is further transformed to phthalic acid by CBA dehydrogenase (PhdK) (28, 36, 37). The *phdK* gene had so far only been found and characterized in *Nocardioides* strain KP7 (28), in which the *phdK* gene was clustered with *phdI* and *phdJ* genes but not with *pht* genes for phthalic acid degradation. The amino acid sequences of the putative PhdK enzyme in strain GO-4 (492 amino acids [aa]) and PhdK in *Nocardioides* strain KP7 (485 aa) shared 40.6% sequence similarity, and both consisted of one domain of the aldehyde dehydrogenase (IPR015590). In *Thalassospira*, homologous genes of *phdI* or *phdJ* genes were not found in their genomes in databases, which is consistent with the fact that strain GO-4 did not grow on 1-hydroxy-2-naphthoic acid. The putative *phdK*-*pht* gene cluster that was found in *Thalassospira* spp. can be considered to be responsible for their ability to grow on CBA as the sole carbon source. To the best of our knowledge, this study is the first report of this putative novel functional gene cluster, whereas further transcriptomic and/or enzymatic examinations are required to fully prove their functions. Although CBA has often been identified as an intermediate product of PAH biodegradation, the bacterial ability to utilize CBA as the sole growth substrate has not been much studied. A previous study reported a *Thalassospira* isolate that was capable of degrading and growing on phthalic acid (38); however, the functional genes that were responsible for the organism’s metabolism were not adequately characterized until the current study. The *phdK-pht* gene cluster was located in a putative foreign gene-rich region of the strain GO-4 genome (Fig. 5) and was found to be shared among genomes of at least 12 other *Thalassospira* strains and even at least two other bacterial genera, *Roseovarius* and *Rhodobium* (Fig. 4). Thus, this gene cluster had likely been acquired in the genomes of these strains by horizontal gene transfers and may have benefited them to inhabit aromatic hydrocarbon-exposed environments.

### Potential of *Thalassospira* to biotransform PAHs.

Comparative genomic analyses of the *Thalassospira* genomes in the current study indicated that homologous genes for an aromatic ring-hydroxylating dioxygenase Pht2/Pht3 and an aromatic ring cleavage dioxygenase PcaGH were commonly found in *Thalassospira* in addition to other genes necessary for further downstream reactions that were clustered with them. However, any related genes of the well-studied *pah*, *phn*, or *nah* genes, which are reported to be responsible for the upstream reactions of phenanthrene/naphthalene degradation and thus are the functional marker genes for PAH degradation (4, 39, 40), were not found in the genomes of strain GO-4 and all other 32 *Thalassospira* strains that were investigated, including strains TSL5-1, *T. xianhensis* P-4^T^, and *T. tepidiphila* 1-1B^T^, which were previously claimed to be potential PAH-degrading strains (13–15). In the literature, there has been no discussion regarding the functional genes or enzymes that were responsible for PAH biotransformation in these strains, and thus these are still open questions. Genomes of these strains are currently all incomplete (27, 100, and 5 uncirculated contigs were deposited for the genomes of strains TSL5-1, P-4^T^, and 1-1B^T^, respectively); therefore, the possibility that other considerable functional genes were overlooked cannot be eliminated. However, according to the genomic overview of *Thalassospira* strains obtained in the current study, members of *Thalassospira* had more likely evolved by adapting to metabolize the downstream, single-ring aromatic acids such as CBA, phthalic acid, and protocatechuic acid. Even if *Thalassospira* strains did not possess the known PAH degradation genes and thus were incapable of growing on PAHs as the sole carbon sources, these PAHs may have been biotransformed by *Thalassospira* through cometabolic reactions (41–43), whereas this phenomenon was not observed in strain GO-4 (see Fig. S1 in the supplemental material).

### Insights into the ecological niches of *Thalassospira* in aromatic hydrocarbon-exposed environments.

**FIG 7:**
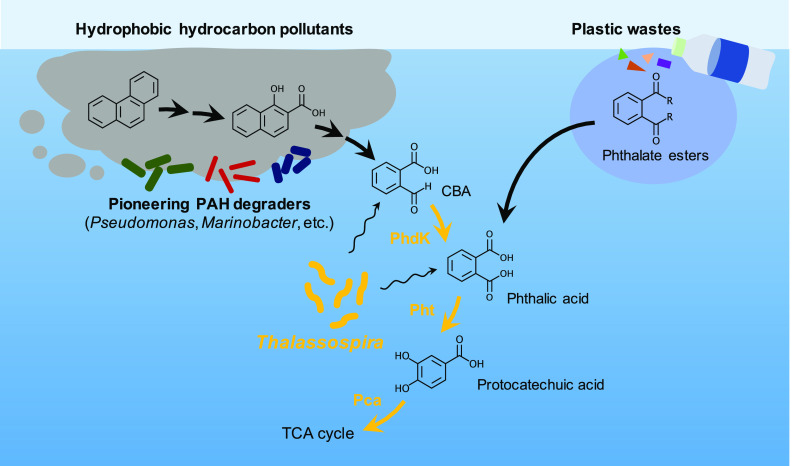
Schematic model summarizing the putative ecological features of *Thalassospira* in aromatic hydrocarbon-exposed marine environments. *Thalassospira* occupied niches that utilize single-ring aromatic acids (CBA, phthalic acid, and protocatechuic acid) that are released from complex, hydrophobic aromatic hydrocarbon pollutants. In PAH-exposed environments, *Thalassospira* likely grew through metabolic dependencies on the pioneering members that possessed specialized enzymes for PAH degradation and thus were responsible for upstream biotransformations. Acquisition of the ability to utilize CBA in *Thalassospira* may have advantaged them to become abundant in PAH-exposed communities, by allowing them to scavenge the relatively less hydrophobic aromatic acids (CBA and phthalic acid) right after becoming available from the more hydrophobic aromatic hydrocarbon sources that originated from ocean oil spill or plastic waste pollution.

Certain microbial groups that are capable of utilizing complex, hydrophobic aromatic compounds like PAHs for their growth and degrading them into more bioavailable compounds are considered to play important roles in nutrient flows in microbial ecosystems, in which other coexisting (secondary) microorganisms grow on metabolites of these pioneers and thus are metabolically dependent upon them (7, 44, 45). Although *Thalassospira* has often been detected as an abundant member of PAH-exposed microbial communities in previous studies (9–12), *Thalassospira* species were not characterized as the pioneering players of PAH degradation. For example, in a previous study by Wang et al. that worked on a phenanthrene-degrading marine bacterial consortium (9), *Thalassospira* was characterized as one of the contributing members that potentially degraded protocatechuate and/or gentisate produced by the pioneer phenanthrene-degrading *Marinobacter* and *Martelella*. Consistent with these previous reports, the current study suggested that members of *Thalassospira* have conserved the ability to grow on single-ring aromatic acids like CBA, phthalic acid, and protocatechuic acid, while lacking known PAH degradation genes; thus, they were more likely metabolically dependent on the coexisting pioneering PAH degraders to grow in PAH-exposed communities. Some members of *Thalassospira* seemed to have acquired the *phdK-pht* genes for CBA and phthalic acid degradation via horizontal gene transfers, in addition to *pca* genes for the downstream protocatechuic acid degradation, which was unexceptionally conserved in *Thalassospira*. Acquisition of the ability to utilize CBA as a growth substrate shall have advantaged *Thalassospira* by allowing them to scavenge these aromatic acids after they became bioavailable from more hydrophobic aromatic hydrocarbon sources (Fig. 7), and hypothetically facilitated *Thalassospira* to commonly inhabit the PAH-exposed environments by outcompeting other microbial groups. Furthermore, the ability to utilize phthalic acid may allow *Thalassospira* to adapt to environments that are polluted with phthalate esters that are derived from plastic wastes (46–48). Strain GO-4 was found to possess at least six different types of esterase-encoding genes; thus, *Thalassosopira* may even contribute to converting phthalate esters to phthalic acid, although no previous study has reported this. *Thalassospira* was also reported to have the abilities of biosurfactant production (49, 50) and biofilm formation (51, 52), which are known as typical strategies to utilize aromatic hydrocarbons (53) and thus also may allow them to dominate aromatic hydrocarbon-degrading microbial communities. Furthermore, *Thalassospira* potentially indirectly contributes to PAH degradation in microbial communities, by mediating synergistic PAH degradation (54) and/or suppressing growth inhibitors of the PAH-degrading pioneers (55) as the other bacterial groups were reported to do in PAH-degrading communities.

In summary, this study provided the first comprehensive functional profile of *Thalassospira* spp. that expanded scientific insight into their potential ecological roles in aromatic hydrocarbon-exposed environments. Further growth assays and metabolite profiling of a new *Thalassospira* isolate strain GO-4 supported the linkage of the metabolic capabilities of strain GO-4 and the results of genomic analyses. In PAH-exposed environments, *Thalassospira* spp. may be adapted to utilize the single-ring aromatic acids (CBA, phthalic acid, and protocatechuic acid) that are released from the degradation of more hydrophobic aromatic substrates, rather than playing a pioneering role in PAH biodegradation. Thus, *Thalassospira* spp. likely co-occupy niches with other pioneer PAH-degrading players that provide them with biodegradation intermediates that may have allowed them to be commonly found as abundant members of microbial communities that developed under exposure to PAHs (9–12). The high culturability of *Thalassospira* in laboratories shall benefit future studies using their pure cultures, which may allow us to discover unknown metabolic functions and to evaluate their potential for biotechnological usages.

## MATERIALS AND METHODS

### Chemicals.

2-Carboxybenzaldehyde (>98% purity) was purchased from Tokyo Chemical Industries (Tokyo, Japan). Phthalic acid (98% purity), *N*,*N*-dimethylformamide (DMF) (>99% purity), methanol (LC-MS grade), and ethyl acetate (high-performance liquid chromatography [HPLC] grade) were purchased from Wako Chemical (Osaka, Japan). 1-Hydroxy-2-naphthoic acid (>97% purity) was purchased from Kanto Chemical Co. (Tokyo, Japan). Phenanthrene (98% purity) was purchased from Sigma-Aldrich (St. Louis, MO, USA).

### Bacterial isolation and growth conditions.

*Thalassospira* sp. strain GO-4 was isolated from a phenanthrene-enriched marine bacterial consortium that originated from the coast of Nojima, Yokohama, Japan (21). The sampling site was not exposed to excess hydrocarbon pollutions such as oil spills, but was surrounded by urban beaches that may face continuous inputs of hydrophobic hydrocarbon pollutants and plastic debris (56). The bacterial consortium was grown on 50 mg L^−1^ phenanthrene as the sole carbon source in Artificial Sea Water medium (57), and bacterial cells were repeatedly transferred to a new medium once per month. Strain GO-4 was isolated from this consortium through dilution-to-extinction techniques using 10 mM glucose as the carbon source, and its purity was confirmed by sequencing analysis of 16S rRNA gene (Macrogen Japan Co., Tokyo, Japan) and microscopic observation using a light microscope (Eclipse E800; Nikon, Tokyo, Japan) or a scanning electron microscope (JSM-6000 NeoScope; JEOL, Tokyo, Japan) after collecting cells on a 0.2-μm-pore polycarbonate membrane filter (Isopore, Merck Millipore, Tullagreen, Ireland). To evaluate the growth capability of strain GO-4 on different carbon sources (phenanthrene, 1-hydroxy-2-naphthoic acid, or 2-carboxybenzaldehyde dissolved in DMF prior to addition), cells were incubated under aerobic conditions with 50 to 100 mg L^−1^ of the selected substrates for ~8 days by rotary shaking at 120 rpm in the dark at 30°C. Bacterial growth was evaluated by visual inspection of turbidity and by measuring the optical density of the cultures at 600 nm (OD_600_).

### Complete genome sequencing of strain GO-4 and comparative genomic analyses.

The complete genome sequence of strain GO-4 was recently announced with detailed methodological information regarding the sequencing analyses and data processing (21). In brief, genomic DNA of strain GO-4 was extracted from a glucose-grown culture by using the NucleoBond high-molecular-weight DNA kit (Macherey-Nagel, Düren, Germany) and was subjected to complete genome sequencing that employed a hybrid assembly of short-read (DNBSEQ-G400; MGI Tech, Shenzhen, China) and long-read (GridION X5; Oxford Nanopore Tech, Oxford, United Kingdom) sequencing technologies. Gene annotation was performed by using the annotation pipelines of NCBI (PGAP v.6.1) and JGI (IMG annotation pipeline v.5.1.6). Comparative genomic analyses using the reference *Thalassospira* genomes deposited in public databases (NCBI and IMG) were conducted as described in a previous work (58); average nucleic acid identity (ANI) among *Thalassospira* strains was determined using FastANI (v.1.32) (59), and sequence alignment comparisons between *Thalassospira* genomes were performed by using Mauve (v.2.4.0) (60).

### Profiling of biotransformation products of strain GO-4 by LC-ESI(−)-MS/MS.

Metabolic products released by strain GO-4 cells that grew on 50 mg L^−1^ CBA were extracted and analyzed by LC-ESI(−)-MS/MS according to Tomiyama et al. (61). Briefly, cultures were extracted with equal volumes of ethyl acetate at pH 7 and pH 2 (pH adjustment by HCl) by liquid-liquid extraction, after which organic phases were filtered through anhydrous sodium sulfate and concentrated *in vacuo* at 28°C via rotary evaporation (Eyela model N-1000; Tokyo Rikakikai Co., Ltd., Tokyo, Japan). Residues were resuspended in 1 mL of methanol and analyzed using a Waters 2690 separations module delivery system in line with a Quattro Ultima triple-stage quadrupole mass spectrometer (Micromass, Manchester, United Kingdom). Extracts were eluted isocratically in 77% methanol/water at a flow rate of 0.3 mL/min and separated on a Shimadzu Shim-pack XR-ODS column (150 mm by 3.0-mm inside diameter, 2.2-μm particle size) that was in line with a Security Guard cartridge system precolumn fitted with a Widepore C_18_ cartridge (Phenomenex, CA). Data were processed by MassLynx software v.4.1.

### Data availability.

The genome sequence of strain GO-4 has been deposited in NCBI GenBank under accession no. CP097807 and in the IMG/MER database under accession no. 2963523905. The raw sequences are available from SRA accession no. SRR19369649 and SRR19369650 under BioProject no. PRJNA841389 and BioSample no. SAMN28591689.
