# Supplementary material for: Comprehensive Genomic Characterization of Marine Bacteria Thalassospira spp. Provides Insights into Their Ecological Roles in Aromatic Hydrocarbon-Exposed Environments
Source: Microbiol Spectr. 2022 Oct 3;10(5):e03149-22. doi: 10.1128/spectrum.03149-22 (PMC9604089; doi:10.1128/spectrum.03149-22)
Supplement: Supplemental file 1 — Supplemental material. Download spectrum.03149-22-s0001.pdf, PDF file, 0.1 MB [file spectrum.03149-22-s0001.pdf]

Supplementary Information

Microbiology Spectrum

Comprehensive genomic characterization of marine bacteria *Thalassospira* spp. provides insights into their ecological roles in aromatic hydrocarbon-exposed environments

Go Kayama, Robert A. Kanaly, and Jiro F. Mori

Graduate School of Nanobiosciences, Yokohama City University, Japan

Corresponding author

Jiro F. Mori

Yokohama City University

morij@yokohama-cu.ac.jp

+81 45 787 2336

Table S1. Detailed information on the short-read and long-read sequencing results.

Table S2. List of the functional genes identified in the putative foreign gene-rich region of the strain GO-4 genome.

Figure S1. Chromatograms of aromatic hydrocarbons extracted from the strain GO-4 cultures after 8 days of incubation.

Table S1. Detailed information on the short-read and long-read sequencing results.

**Number of reads**		**Average read length (bp)**		**Total read size (bp)**		**Assembly total size (bp)**	**Assembly GC content (%)**	**Assembly coverage**
DNBSEQ (short-read)	GridION (long-read)	DNBSEQ (short-read)	GridION (long-read)	DNBSEQ (short-read)	GridION (long-read)			
22,517,128	68,243	200	17,703	4,503,425,600	1,208,089,891	4,546,452	54.8	1,256

Table S2. List of the functional genes identified in the putative foreign gene-rich region of the strain GO-4 genome.

Gene	EC	AA length	Locus	IMG Gene ID
**Nitrate reduction**				
Nitrate/nitrite transporter NarK		889	3957161-3959830	2963527598
Nitrate reductase alpha subunit NarG	1.7.5.1	1246	3959898-3963638	2963527599
Nitrate reductase beta subunit NarH	1.7.5.1	507	3963635-3965158	2963527600
Nitrate reductase delta subunit NarJ		234	3965158-3965862	2963527601
Nitrate reductase gamma subunit NarI	1.7.5.1	231	3965874-3966569	2963527602
**Nitrous oxide reduction**				
Nitrous oxidase accessory protein NosD		446	3977215-3978555	2963527616
Nitrous oxide reductase NosZ	1.7.2.4	649	3978559-3980508	2963527617
Nitrous oxide reductase transcriptional regulator NosR		755	3980505-3982772	2963527618
**Isoquinoline degradation**				
Isoquinoline 1-oxidoreductase alpha subunit IorA	1.3.99.16	182	4004972-4005520	2963527638
Isoquinoline 1-oxidoreductase beta subunit IorB	1.3.99.16	723	4005524-4007695	2963527639
**Ribose transport system**				
Ribose transport system substrate-binding protein RbsB		370	4041990-4043102	2963527667
Ribose transport system ATP-binding protein RbsA	7.5.2.7	537	4043129-4044742	2963527668
Ribose transport system permease protein RbsC		341	4044739-4045764	2963527669
Ribose transport system permease protein RbsC		333	4045773-4046774	2963527670
Ribose transport system substrate-binding protein RbsB		331	4050183-4051178	2963527674
Ribose transport system substrate-binding protein RbsB		315	4052404-4053351	2963527676
Ribose transport system ATP-binding protein RbsA	7.5.2.7	505	4053454-4054971	2963527677
Ribose transport system permease protein RbsC		327	4054975-4055958	2963527678
Ribose transport system permease protein RbsC		333	4056784-4057785	2963527680

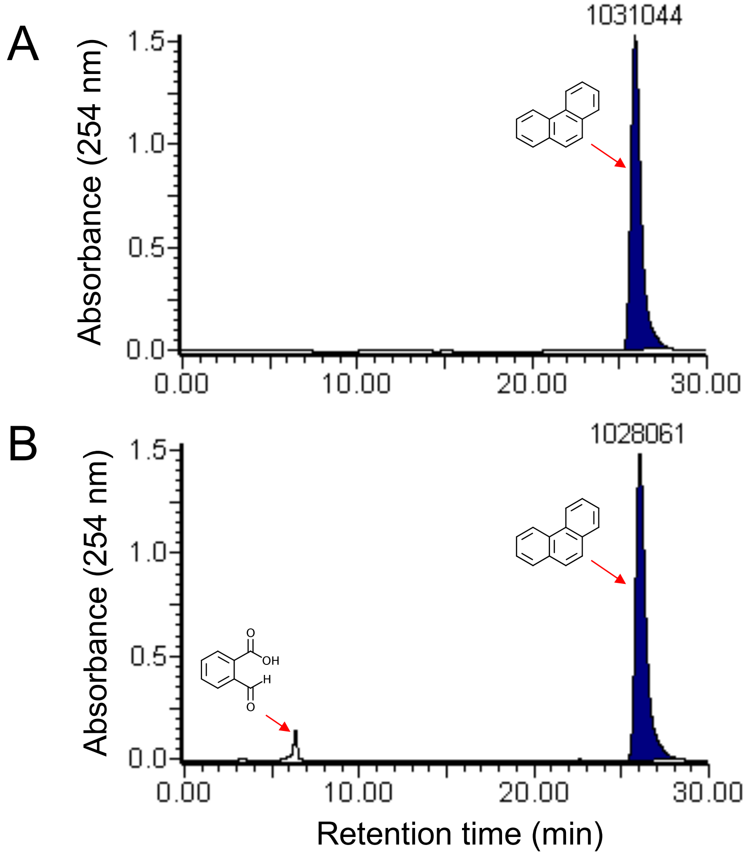

Figure S1. Chromatograms of aromatic hydrocarbons extracted from the strain GO-4 cultures after 8 days of incubation: **(A)** strain GO-4 cells exposed to 2-carboxybenzaldehyde and phenanthrene in the same culture and **(B)** the abiotic control culture without bacterial cells. Peak areas for the phenanthrene peaks were shown.
